# Vaccination in dermatology 2025: Update considering current recommendations of the German Standing Committee on Vaccination

**DOI:** 10.1111/ddg.15785

**Published:** 2025-06-11

**Authors:** Johanna Stoevesandt, Marc Schmalzing, Sophia Mohme, Matthias Goebeler

**Affiliations:** ^1^ Klinik und Poliklinik für Dermatologie Venerologie und Allergologie University Hospital Würzburg Würzburg Germany; ^2^ Medizinische Klinik und Poliklinik II Rheumatologie/Klinische Immunologie University Hospital Würzburg Würzburg Germany; ^3^ Hautarztpraxis Dr. Mohme Porta Westfalica Germany

**Keywords:** COVID‐19, herpes zoster, high‐dose vaccine, immunosuppression, influenza, Mpox, pneumococci, respiratory syncytial virus

## Abstract

The immunosuppressive and immunomodulatory treatment of dermatological patients necessitates the regular review and updating of standard vaccinations and vaccines indicated for specific conditions. The *German Standing Committee on Vaccination* (STIKO) at the *Robert Koch Institute* regularly publishes evidence‐based vaccination recommendations, which are adapted to the current epidemiological situation and availability of vaccines. Since 2020, several changes have been made that are relevant for patients with dermato(onco)logical diseases: *(1)* COVID‐19 was defined as a new viral disease and several vaccines have been introduced; *(2)* in response to the global Mpox outbreak in 2022, a non‐replicating live vaccine based on the modified Ankara vaccinia virus, which was approved in 2013 for the prevention of smallpox, was given an indication extension; *(3)* a new inactivated high‐dose vaccine was approved for influenza vaccination of persons aged 60 years and older; *(4)* a new 20‐valent conjugate vaccine is available for pneumococcal vaccination; *(5)* two recombinant vaccines against the respiratory syncytial virus (RSV) were recently approved. This article discusses the correspondingly adapted STIKO recommendations for adults, with particular emphasis on their implementation in immunocompromised patients in dermatology.

## INTRODUCTION

The prescription of immunosuppressive and immunomodulating agents, e.g. for the treatment of psoriasis, atopic dermatitis or autoimmune blistering dermatoses, as well as the treatment of patients with advanced dermatologic tumors, requires specific vaccination recommendations. In addition, several diseases that can be prevented by vaccination – particularly herpes zoster and, occasionally, other viral exanthems – are managed by dermatologists. In 2020, Mohme et al. published comprehensive vaccination recommendations relevant to dermatology in the *JDDG: Journal der Deutschen Dermatologischen Gesellschaft*, considering the impact of commonly used immunosuppressive and immunomodulatory therapies.^1^ Since then, there have been significant changes, not least as a result of the COVID‐19 pandemic and a global Mpox outbreak, as well as the approval of new vaccines. This update focuses on the adapted recommendations for adults from the *German Standing Committee on Vaccination* (STIKO) at the *Robert Koch Institute* (RKI), with special emphasis on implementation in immunocompromised dermatological patients.^2^ Table 1 summarizes the recommendations valid as of 01/2025. Complete vaccination recommendations for adults (Table S1; online Supplement only) and an overview of the most important vaccinations for immunocompromised children and adolescents (Table S2; online Supplement only) are available as an online supplement.

**TABLE 1:** Vaccination recommendations for immunocompetent and immunocompromised/chronically ill adults.^*^

Immunocompetent adults	Immunocompromised/chronically ill adults	Available vaccines^**^; vaccination schedules; comments
***COVID‐19***		
*Standard vaccination* of adults including pregnant women from the 2nd trimester with incomplete baseline immunity; annual vaccination of persons aged 60 years and over; *annual vaccination* of close contacts of immunodeficient persons and residents of care facilities; annual *occupational vaccination* of healthcare workers	Annual booster vaccination of people with immunosuppression and/or other risk factors (e.g. chronic respiratory, liver, kidney, cardiovascular or CNS disease, advanced tumors, diabetes mellitus, obesity, trisomy 21); in severe immunosuppression, additional vaccinations and serological testing may be required	mRNA (Comirnaty^TM^, Spikevax^TM^) and protein‐based (e.g. Nuvaxovid^TM^) vaccines, each with the variant adaptation recommended by the WHO; baseline immunity is achieved by at least 3 antigen contacts, of which at least 1 vaccination; the indication groups mentioned receive an annual booster in autumn
***Herpes zoster***		
*Standard vaccination* of persons aged 60 years and older	Vaccination of persons aged 50 years and older with congenital/acquired immunodeficiency or chronic disease (e.g. HIV infection, rheumatoid arthritis, systemic lupus erythematosus, chronic inflammatory bowel disease, respiratory disease, diabetes mellitus); considerations not yet covered by STIKO recommendations include vaccination of persons aged ≥ 18 years taking JAK inhibitors and passive immunization of patients with herpes zoster at increased risk of severe outcome with varicella‐zoster immunoglobulin^20^	Recombinant, adjuvanted vaccine (Shingrix^TM^); two doses at 2 to 6 months interval; vaccination of adults under 50 years is not covered by STIKO recommendations (cost coverage to be clarified)
***Influenza***		
*Standard vaccination* of persons aged 60 years and over; vaccination of pregnant women from the second trimester (or first trimester in case of increased risk), household contacts of immunocompromised persons, residents of old people's homes and nursing homes, and in the event of an imminent epidemic (if recommended by health authorities); *occupational vaccination* of medical staff and persons with extensive public contact or contact with wild birds	Vaccination in cases of increased risk due to chronic disease (e.g. respiratory, cardiovascular, neurological or renal disease, diabetes mellitus) or congenital/acquired immunodeficiency, including immunosuppressive therapy	Inactivated vaccines (e.g. Influsplit^TM^, Influvac^TM^), high‐dose vaccines (Efluelda^TM^), adjuvanted vaccines (Fluad^TM^); annual vaccination in autumn with a vaccine containing the current WHO‐recommended antigen combination; people aged 60 years and over receive the high‐dose vaccine or adjuvanted vaccine regardless of their immune status
***Mpox (formerly monkeypox)***		
No standard vaccination, *pre‐exposure vaccination* of men with sexual contact with changing male partners; *occupational vaccination* of laboratory staff working with infectious orthopox viruses; *post‐exposure vaccination* after close physical contact or prolonged face‐to‐face contact with infected persons, medical personnel after close contact without adequate protective equipment, laboratory staff after unprotected contact with infectious material	Immunocompromised patients with an indication for vaccination will receive 2 doses of vaccine regardless of their smallpox vaccination status and will be given priority in the event of a supply shortage	Non‐replicating live vaccine (Imvanex^TM^), based on modified Ankara vaccinia virus; 2 subcutaneous vaccine doses given at least 28 days apart; 1 vaccine dose is sufficient for immunocompetent individuals with a history of previous smallpox vaccination; *post‐exposure vaccination* of asymptomatic persons should be initiated no later than 14 days after exposure to Mpox
***Pneumococci***		
*Standard vaccination* of persons aged 60 and over, *occupational vaccination* in case of exposure to metal fumes	Vaccination of adults with congenital or acquired immunodeficiency, on immunosuppressive therapy or with chronic diseases (e.g. cardiovascular, respiratory, metabolic, malignant) or with local risk factors for pneumococcal meningitis (e.g. cochlear implant, cerebrospinal fluid fistula)	20‐valent conjugate vaccine PCV‐20 (Prevenar 20^TM^); administration of one dose even if previously vaccinated with other pneumococcal vaccines; the minimum interval of 6 years since the last dose of PPSV‐23 may be reduced to 1 year in severe immunodeficiency; the minimum interval since the last dose of PCV‐13 is 1 year
***Respiratory syncytial virus (RSV)***		
*Standard vaccination* of people aged 75 and over; *vaccination* of residents of care facilities aged ≥ 60 years	Vaccination of adults aged ≥ 60 years ‐ with severe congenital or acquired immunodeficiency ‐ with severe chronic illness (e.g. respiratory disease, cardiovascular or kidney disease, haemato‐oncological disease, diabetes mellitus with complications)	Two recombinant, protein‐based vaccines (monovalent/adjuvanted: Arexvy^TM^; bivalent/non‐adjuvanted: Abrysvo^TM^); single dose vaccination before the next RSV season

* Table 1 is limited to the vaccinations discussed in the text; see online supplementary Table S1 for a complete summary of current vaccination recommendations for immunocompetent and immunocompromised adults and online supplementary Table S2 for the implementation of the discussed indication vaccinations in children and adolescents

** A comprehensive list of available vaccines can be found on the website of the Paul‐Ehrlich‐Institute (https://www.pei.de/DE/arzneimittel/impfstoffe/impfstoffe‐node.html)

A reorganization of personnel in 2024 also resulted in changes within the STIKO itself. Additional experts from the fields of communication and modelling were appointed and the maximum term of office was limited to three three‐year periods.

## VACCINATION AS A WAY OUT OF THE PANDEMIC: NEW VACCINATION RECOMMENDATIONS FOR NEW VIRUSES

### SARS‐CoV‐2/COVID‐19

The COVID‐19 pandemic has prompted the development, clinical testing and global distribution of new vaccines at an unprecedented speed.^3^ The STIKO currently recommends the use of mRNA‐ or protein‐based vaccines, which are regularly updated according to the variant adaptation recommended by the *World Health Organization* (WHO). Baseline immunity to SARS‐CoV‐2 should be established in all adults.^2^ This is achieved by three exposures to SARS‐CoV‐2 antigen, at least one of which should be to a vaccine. An annual booster vaccination in autumn is recommended for individuals at increased risk of serious outcomes and those with an increased likelihood of infection or transmission, including healthcare workers (Table 1). In immunocompromised patients with a severely impaired immune response (e.g. after organ transplantation), additional doses of vaccine may be required at intervals of at least 4 weeks to achieve baseline immunity;^2^, ^4^, ^5^ the effect is monitored serologically by detecting specific antibodies to the SARS‐CoV‐2 spike protein. If an adequate immune response is not achieved despite repeated vaccination, the off‐label use of increased vaccine doses or a change in preparation may be considered.^4^ In patients with severe immunosuppression, several booster vaccinations per year may be advisable to maintain protection.^2^ Additional vaccine doses may also be considered during treatment with rituximab.^6^ However, the recommendation that all vaccines should ideally be given 4 weeks before or at least 6 months after administration of rituximab seems to be more important. The antibody combination tixagevimab/cilgavimab (Evusheld^TM^) was approved in 2022 for passive immunization or pre‐exposure prophylaxis. However, according to the updated STIKO recommendation of February 2023, it should only be used in selected individual cases due to a reduced neutralizing capacity with regard to the circulating Omicron sub‐variants. The administration of tixagevimab/cilgavimab can be considered as an additional preventive measure in the context of B‐cell depleting therapy.^7^

### Mpox

An increasing number of cases of Mpox (formerly monkeypox) have also been recorded in Germany in 2022, as a manifestation of a global outbreak of clade II Mpox.^8^ Dermatology clinics and practices, especially in urban areas, are often the first port of call for Mpox. Dermatologists should therefore include the disease in their differential diagnosis and be familiar with treatment and vaccination recommendations.^9^ Imvanex^TM^, a live vaccine based on the modified Ankara vaccinia virus, which cannot replicate in humans, has been licensed in the *European Union* for the prevention of smallpox since 2013 and received an extension for Mpox in 2022. If not available, the vaccine Jynneos^TM^ from the same manufacturer can be used. The STIKO recommends post‐exposure Mpox vaccination of unvaccinated adults with sexual contact or prolonged unprotected face‐to‐face contact with infected persons (Table 1).^2^, ^10^ This recommendation also applies to medical personnel if they have been exposed without adequate protective equipment (face mask and gown). In addition, the STIKO advises pre‐exposure vaccination of persons with an increased risk of infection.^10^ This includes adult men who report sexual contact with frequently changing male partners. Primary vaccination consists of two subcutaneous doses given at least 28 days apart. One dose is considered sufficient for immunocompetent individuals who have been vaccinated against smallpox in the past. Vaccination of immunocompromised patients and, unlike with previous smallpox vaccines capable of replication in humans, also patients with atopic eczema is recommended if indicated.^11^ Immunodeficient patients receive two doses of vaccine regardless of whether they have had a previous smallpox vaccination. They should be prioritized for vaccination in the event of supply shortages due to the increased risk of severe disease.^10^, ^12^

The situation has changed with the East African outbreak of clade Ib Mpox, which is considered more virulent than clade II. This led the WHO to declare a *Public Health Emergency of International Concern* in August 2024; isolated cases have been reported outside the African continent. Available vaccines are considered effective.

## UPDATE ON THE MOST IMPORTANT VACCINATIONS FOR IMMUNOCOMPROMISED PATIENTS: INFLUENZA, PNEUMOCOCCI, HERPES ZOSTER

### Influenza

The German STIKO recommends annual vaccination in autumn with an inactivated influenza vaccine adapted to the antigen combination proposed by the WHO for people aged 60 years and over, pregnant women, residents of care facilities, chronically ill/immunocompromised people and their contacts (Table 1). Vaccination is also recommended for healthcare workers and other groups at increased occupational risk of infection. Since 2021, the STIKO has recommended an inactivated high‐dose vaccine for people aged 60 years and over^13^ that contains four times the amount of antigen (60 µg instead of 15 µg) as a conventional preparation. According to a STIKO recommendation from October 2024, an MF‐59 adjuvanted vaccine can be used as an alternative for this age group. This age‐specific recommendation is based on the increased likelihood of hospitalization and severe outcomes in older people, combined with a reduced immune response to the flu vaccine, which is partly due to immune senescence.^14^ The use of the high‐dose vaccine or adjuvanted vaccine results in a small but statistically significant reduction in laboratory‐confirmed influenza infections compared with conventional preparations. The reactogenicity in terms of vaccine‐related local and mild systemic reactions is considered to be slightly increased.^13^ In line with WHO recommendations, the STIKO has been advising the use of trivalent instead of tetravalent influenza vaccines since August 2024. These do not include the B/Yamagata lineage antigen, which has decreased strongly in global circulation. Trivalent inactivated influenza vaccines are expected to be available in Europe for the 2025/2026 season.

### Pneumococci

One dose of the 20‐valent conjugate vaccine (PCV‐20) Prevenar 20^TM^ is recommended for immunocompetent individuals aged 60 years and older as well as adults with occupational exposure to metal fumes, and chronically ill/immunocompromised adult patients regardless of age (Table 1). The use of PCV‐20 was introduced in 2023, replacing recommendations to administer the 23‐valent polysaccharide vaccine (PPSV‐23) in immunocompetent individuals or the 13‐valent conjugate vaccine (PCV‐13) followed by PPSV‐23 in immunocompromised patients.^15^ The change in recommendation was justified by the superior immunogenicity of PCV‐20 compared with PPSV‐23 and its broader serotype coverage compared with PCV‐13. Comparative data on clinical effectiveness are not available.^15^, ^16^ PCV‐20 can be co‐administered with an influenza vaccine or an RNA‐based COVID‐19 vaccine;^17^ data on the need for booster vaccinations are not yet available. In March 2024, Prevenar 20^TM^ received an extension of approval for children ≥ 6 weeks; however, the STIKO continues to recommend the use of PCV‐13/PCV‐15 (primary vaccination from 2 months) and, if necessary, PPSV‐23 (chronically ill persons between 2 and 17 years).

### Herpes zoster

Vaccination against herpes zoster is recommended for all persons aged 60 years and older and for chronically ill/immunocompromised patients from the age of 50 years (Table 1). Recent data suggest that the vaccine may have a protective effect against dementia.^18^ In addition to the current STIKO recommendations, the *German Society of Rheumatology and Clinical Immunology* (DGRh) advises adult patients taking a Janus kinase (JAK) inhibitor (e.g. baricitinib, tofacitinib, upadacitinib) to be vaccinated from the age of 18 years (https://dgrh.de/Start/Versorgung/Therapieinformationen/Therapieinformationsbögen.html). This recommendation is based on the increased risk of zoster virus reactivation, which varies depending on the JAK inhibitor used.^19^ It is transferable to the use of JAK inhibitors for dermatological indications. It should be noted, however, that the vaccination of adults under 50 years of age is not included in the vaccination guidelines of the *German Federal Joint Committee* (https://www.g‐ba.de/richtlinien/60/) and is therefore not generally eligible for reimbursement despite approval.

Recent considerations, which also go beyond the STIKO recommendations, concern passive immunization of chronically ill patients with acute herpes zoster infection, if there is a risk of severe outcome or systemic spread of the virus. Several older studies and a small recent case series^20^ suggest that adding varicella‐zoster immunoglobulin to standard antiviral therapy may have a beneficial effect on the course of herpes zoster. An observational study (EUPAS 103611) targeted to enroll 120 patients treated with varicella‐zoster immunoglobulin and 40 controls was initiated by the manufacturer in June 2023.

## NEW VACCINATION OPTION FOR A WELL‐KNOWN PATHOGEN: RESPIRATORY SYNCYTIAL VIRUS

The respiratory syncytial virus (RSV), which is widespread throughout the world, causes respiratory disease at any age. Severe and sometimes life‐threatening courses are most common in infants and young children, especially when co‐infected with bacteria, fungi or other viruses, but also in the elderly, the chronically ill and the immunocompromised.^21^ With the recombinant vaccines Arexvy^TM^ (monovalent, adjuvanted) and Abrysvo^TM^ (bivalent), two preparations have been available for vaccination since 2023. The adjuvanted vaccine is approved for people aged 50 and over, while Abrysvo^TM^ is also approved for pregnant women in order to provide passive protection for their newborns/infants. Since August 2024, the STIKO has recommended a single standard vaccination for people aged 75 and over. Patients with congenital or acquired immunodeficiency or severe chronic diseases (e.g. affecting the respiratory organs, kidneys, cardiovascular system, or hematopoietic system) and residents of care facilities are recommended to be vaccinated against RSV from the age of 60 (Table 1).^22^ Palivizumab and, since 2022, nirsevimab are two monoclonal antibodies directed against the viral F protein available for RSV prophylaxis in infants and young children. In its decision from June 2024, the STIKO recommends a single passive immunization of newborns/infants with nirsevimab before the start of their first RSV season.^23^

## DEALING WITH VACCINATION HESITANCY

Triggered by the rapid development of new vaccines and the global vaccination campaign during the COVID‐19 pandemic, vaccination has become the subject of personal and political disputes between pro‐vaccinators, anti‐vaccinators, and many who are hesitant.^24^ Patients with dermatological conditions and/or planned immunosuppressive treatment are also often reluctant to receive necessary vaccinations.^25^ The RKI provides so‐called “truth sandwiches” for educational dialogue, which specifically address common vaccination myths and misinformation and explain them with factual information (https://www.rki.de/EN/Topics/Infectious‐diseases/Immunisation/Information‐material/Vaccination‐myths/effectively‐debunking‐misinformation‐node.html).

## CONCLUSIONS

The immunosuppressive and immunomodulatory treatment of dermatological patients requires regularly review and updating of the necessary standard vaccinations and vaccines for specific indications.^1^ Dermatologists should be familiar with the current STIKO recommendations (Table 1) and be aware that in the event of a change in the epidemiological situation and/or the development of new vaccines, adjustments will be made regularly and summarized once a year in the *Epidemiological Bulletin* published by the RKI.^2^

## CONFLICT OF INTEREST STATEMENT

None.

## Supporting information



Supporting information
