# Supplementary material for: Vaccination in dermatology 2025: Update considering current recommendations of the German Standing Committee on Vaccination
Source: J Dtsch Dermatol Ges. 2025 Jun 11;23(8):925–30. doi: 10.1111/ddg.15785 (PMC12338432; doi:10.1111/ddg.15785)
Supplement: Supplementary file 1 — Supporting information [file DDG-23-925-s001.docx]

Supplemental Material (Tables S1 and S2)

Vaccination in dermatology 2025: update considering current recommendations of the German Standing Committee on Vaccination

Johanna Stoevesandt^1^, Marc Schmalzing^2^, Sophia Mohme^1,3^, Matthias Goebeler^1^

^1^Klinik und Poliklinik für Dermatologie, Venerologie und Allergologie,
Universitätsklinikum Würzburg, Würzburg

^2^Medizinische Klinik und Poliklinik II, Rheumatologie/Klinische Immunologie, Universitätsklinikum Würzburg, Würzburg

^3^Hautarztpraxis Dr. Mohme, Porta Westfalica

## **Table S1** Vaccination recommendations for immunocompetent and immunocompromised/chronically ill adults

| **Immunocompetent adults** | **Immunocompromised/chronically ill adults** | **Available vaccines; vaccination schedules; comments** |
| --- | --- | --- |
| COVID-19 |  |  |
| **Standard vaccination of adults including pregnant women from the 2nd trimester with incomplete baseline immunity; annual vaccination of persons aged 60 years and over; annual vaccination of close contacts of immunodeficient persons and residents of care facilities; annual occupational vaccination of healthcare workers** | **Annual booster vaccination of people with immunosuppression and/or other risk factors (e.g. chronic respiratory, liver, kidney, cardiovascular or CNS disease, advanced tumours, diabetes mellitus, obesity, trisomy 21); in severe immunosuppression, additional vaccinations and serological testing may be required** | **mRNA (Comirnaty^TM^, Spikevax^TM^) and protein-based (e.g. Nuvaxovid^TM^) vaccines, each with the variant adaptation recommended by the WHO; baseline immunity is achieved by at least 3 antigen contacts, of which at least 1 vaccination; the indication groups mentioned receive an annual booster in autumn** |
| Diphtheria |  |  |
| Catch-up vaccination of persons with missing or incomplete primary vaccination; subsequent booster doses every 10 years; post-exposure vaccination after close face-to-face contact with infected persons if the last vaccination was > 5 years ago | As recommended for immunocompetent individuals | Diphtheria toxoid; primary immunisation is achieved by 3 vaccine doses; adults receive the next due booster as a Tdap or Tdap-IPV combination if necessary |
| Tick-borne encephalitis (TBE) |  |  |
| No standard vaccination; vaccination of persons exposed to ticks in areas at risk for TBE; occupational vaccination of exposed laboratory personnel and forestry workers or farmers in risk areas | As recommended for immunocompetent individuals | Inactivated TBE virus; primary vaccination series and subsequent boosters according to product-specific prescribing information; **the** **list of TBE risk areas is updated regularly** |
| Haemophilus influenzae B |  |  |
| No standard adult vaccination | Vaccination in cases of anatomical or functional asplenia (e.g. sickle cell disease) | Conjugate vaccine; single-dose vaccination where indicated; procurement of monovalent vaccines through international pharmacies |
| Hepatitis A |  |  |
| No standard vaccination, vaccination of individuals with increased sexual exposure, intravenous drug users, residents of psychiatric facilities; occupational vaccination of healthcare workers, nursery/residential home staff, workers exposed to wastewater; vaccination before travelling to endemic areas; post-exposure vaccination, especially for outbreaks in community settings | Vaccination of patients with chronic diseases that require frequent transfusions of blood components (e.g. haemophilia) or liver disease | Inactivated hepatitis A virus; monovalent vaccines and combinations with recombinant hepatitis B vaccine; primary vaccination and, if necessary, booster doses according to product-specific prescribing information; post-exposure vaccination with monovalent vaccine within 14 days after exposure |
| Hepatitis B |  |  |
| No standard adult vaccination;  Vaccination in case of increased exposure (e.g. household contacts of hepatitis B antigen carriers, prisoners, residents of psychiatric institutions); occupational vaccination, e.g. of healthcare workers, police officers, staff in institutions with an increased number of hepatitis B-infected persons; travel vaccination based on individual risk assessment; post-exposure vaccination of inadequately vaccinated individuals following needlestick injury or blood contact with infectious material (if necessary, together with hepatitis B immunoglobulin) | Vaccination of patients who are expected to have a severe course due to immunodeficiency, immunosuppressive therapy or chronic disease (e.g. HIV, hepatitis C, renal insufficiency requiring dialysis); the indication for vaccination should be based on the actual risk of exposure | Various recombinant vaccines; primary vaccination series according to product-specific prescribing information; those vaccinated as children have a booster dose in case of high exposure; serological testing is indicated 4-8 weeks after completion of the vaccination series, revaccination is recommended for low-responders and non-responders; serological testing and, if necessary, booster vaccination are also recommended for successfully vaccinated individuals in case of humoral immunodeficiency (1x/year) or increased occupational/private exposure (first time 10 years after vaccination) |
| Herpes zoster |  |  |
| Standard vaccination of persons aged 60 years and older | Vaccination of persons aged 50 years and older with congenital/acquired immunodeficiency or chronic disease (e.g. HIV infection, rheumatoid arthritis, systemic lupus erythematosus, chronic inflammatory bowel disease, respiratory disease, diabetes mellitus); **considerations not yet covered by STIKO recommendations include vaccination of persons aged ≥18 years taking JAK inhibitors and passive immunisation of patients with herpes zoster at increased risk of severe outcome with varicella-zoster immunoglobulin** | Recombinant, adjuvanted vaccine (Shingrix^TM^); two doses at 2 to 6 months interval; vaccination of adults under 50 years is not covered by STIKO recommendations (cost coverage to be clarified) |
| Human papillomavirus (HPV) |  |  |
| No standard adult vaccination; adults ≥ 18 years may benefit from vaccination, although effectiveness is reduced in non-HPV-naive individuals | E.g. vaccination of adults with HIV infection or systemic lupus erythematosus | Recombinant vaccines; vaccination according to product-specific prescribing information; vaccination of adults outside the official STIKO recommendations (cost coverage to be clarified) |
| Influenza |  |  |
| Standard vaccination of persons aged 60 years and over; vaccination of pregnant women from the second trimester (or first trimester in case of increased risk), household contacts of immunocompromised persons, residents of old people's homes and nursing homes, and in the event of an imminent epidemic (if recommended by health authorities); occupational vaccination of medical staff and persons with extensive public contact or contact with wild birds | Vaccination in cases of increased risk due to chronic disease (e.g. respiratory, cardiovascular, neurological or renal disease, diabetes mellitus) or congenital/acquired immunodeficiency, including immunosuppressive therapy | Inactivated vaccines (e.g. Influsplit^TM^, Influvac^TM^), **high-dose vaccines (Efluelda^TM^), adjuvanted vaccines (Fluad^TM^)**; annual vaccination in autumn with a vaccine containing the current WHO-recommended antigen combination; **people aged 60 years and over receive the high-dose vaccine or adjuvanted vaccine regardless of their immune status** |
| Measles |  |  |
| Catch-up vaccination of adults born after 1970 with unclear vaccination status, without vaccination or with only one vaccination; mandatory vaccination (from 1 March 2020 under § 20, German Infection Protection Act) for admission to or attendance of a public institution (e.g. kindergarten, school); outbreak-related vaccination; occupational vaccination of persons born after 1970 who work in healthcare facilities such as hospitals and medical practices or in community facilities/housing (mandatory from 1 March 2020), and for work involving contact with potentially infectious material; post-exposure vaccination of contacts who are not or not reliably immune | Live vaccines are contraindicated in immunosuppressed patients (depending on the active substance and dose); susceptible immunocompromised patients and pregnant women receive post-exposure prophylaxis with standard immunoglobulins after contact with measles patients (caution: the success of subsequent MMR vaccination is limited in the 8 months after immunoglobulin administration) | Various live attenuated vaccines, all as combination vaccines (MMR, MMRV); one dose of vaccine is required for catch-up and outbreak vaccination of adults; two documented doses are required for the other indications |
| Meningococci |  |  |
| No standard adult vaccination; occupational vaccination of exposed laboratory personnel; outbreak-related vaccination as recommended by health authorities; travel vaccination for endemic areas; post-exposure vaccination of unvaccinated close contacts | Vaccination (serogroups A, C, W, Y, and B) of patients with congenital or acquired immunodeficiency, in particular with  - complement component or properdin deficiency  - treatment with C5 complement inhibitors  - hypogammaglobulinaemia  - anatomical or functional asplenia (e.g. sickle cell disease) | Conjugate vaccines against serogroups A, C, W and Y; recombinant protein-based vaccines against serogroup B; vaccination according to product-specific prescribing information |
| Mpox (formerly "monkeypox") |  |  |
| **No standard vaccination, pre-exposure vaccination of men with sexual contact with changing male partners; occupational vaccination of laboratory staff working with infectious orthopox viruses; post-exposure vaccination after close physical contact or prolonged face-to-face contact with infected persons, medical personnel after close contact without adequate protective equipment, laboratory staff after unprotected contact with infectious material** | **Immunocompromised patients with an indication for vaccination will receive 2 doses of vaccine regardless of their smallpox vaccination status and will be given priority in the event of a supply shortage** | **Non-replicating live vaccine (Imvanex^TM^), based on modified Ankara vaccinia virus; 2 subcutaneous vaccine doses given at least 28 days apart; 1 vaccine dose is sufficient for immunocompetent individuals with a history of previous smallpox vaccination; post-exposure vaccination of asymptomatic persons should be initiated no later than 14 days after exposure to Mpox** |
| Mumps |  |  |
| Occupational vaccination of persons born after 1970 who work in healthcare facilities such as hospitals and medical practices or in community facilities/housing, and for work involving contact with potentially infectious material; post-exposure vaccination of contacts who are not or not reliably immune | Live vaccines are contraindicated in immunosuppressed patients (depending on the active substance and dose) | Various live attenuated vaccines, all as combination vaccines (MMR, MMRV); two vaccine doses are required |
| Pertussis |  |  |
| Single booster vaccination in adulthood; vaccination of pregnant women at the beginning of the third trimester and other persons with close contact to newborns (every 10 years); occupational vaccination of medical staff and persons working in community facilities (every 10 years) | As recommended for immunocompetent individuals | Acellular component vaccines; monovalent vaccines are not available, the booster dose in adulthood is given as a Tdap combination at the time of the next tetanus/diphtheria vaccination due, or as a Tdap-IPV combination if indicated |
| Pneumococci |  |  |
| Standard vaccination of persons aged 60 and over, occupational vaccination in case of exposure to metal fumes | Vaccination of adults with congenital or acquired immunodeficiency, on immunosuppressive therapy or with chronic diseases (e.g. cardiovascular, respiratory, metabolic, malignant) or with local risk factors for pneumococcal meningitis (e.g. cochlear implant, cerebrospinal fluid fistula) | **20-valent conjugate vaccine PCV-20 (Prevenar 20^TM^); administration of one dose even if previously vaccinated with other pneumococcal vaccines; the minimum interval of 6 years since the last dose of PPSV-23 may be reduced to 1 year in severe immunodeficiency; the minimum interval since the last dose of PCV-13 is 1 year** |
| Poliomyelitis |  |  |
| Booster vaccination in adolescence or adulthood (and catch-up vaccinations if the primary series has been missed); vaccination of refugees/asylum seekers from areas with a risk of infection; travel vaccination for endemic areas; occupational vaccination of medical staff with potential contact to infected persons, staff in facilities for refugees/asylum seekers, laboratory personnel with potential risk of infection; post-exposure vaccination of all contacts, regardless of vaccination status | As recommended for immunocompetent individuals | Inactivated poliovirus (IPV); monovalent and combination vaccines are available; adults are considered fully vaccinated if they have received a primary vaccination series (3 doses) and an additional booster; occupational vaccination is boostered every 10 years if exposure continues |
| Respiratory syncytial virus (RSV) |  |  |
| **Standard vaccination of people aged 75 and over; vaccination of residents of care facilities aged ≥ 60 years** | **Vaccination of adults aged ≥ 60 years**  **- with severe congenital or acquired immunodeficiency**  **- with severe chronic illness (e.g. respiratory disease, cardiovascular or kidney disease, haemato-oncological disease, diabetes mellitus with complications)** | **Two recombinant, protein-based vaccines (monovalent/adjuvanted: Arexvy^TM^; bivalent/non-adjuvanted: Abrysvo^TM^); single dose vaccination before the next RSV season** |
| Rubella |  |  |
| Occupational vaccination (as recommended for mumps); vaccination of women of childbearing age with unclear vaccination status, without vaccination or with only one vaccination | Live vaccines are contraindicated in immunosuppressed patients (depending on the active substance and dose) | Various live attenuated vaccines, all as combination vaccines (MMR, MMRV); two vaccine doses are required |
| Tetanus |  |  |
| Catch-up vaccination of persons with missing or incomplete primary vaccination; subsequent booster doses every 10 years; post-exposure vaccination for injuries/wounds | As recommended for immunocompetent individuals | Tetanus toxoid; see schedule for diphtheria; post-exposure vaccination may be combined with tetanus immunoglobulin (depending on vaccination status and type of wound) |
| Varicella |  |  |
| Vaccination of seronegative women planning a pregnancy, of susceptible individuals (i.e. with a negative varicella history or seronegative) with severe atopic dermatitis and susceptible contacts of those at risk (e.g. pregnant women, immunocompromised individuals, neonates); occupational vaccination of seronegative persons who work in healthcare facilities such as hospitals and medical practices or in community facilities/housing, and for work involving contact with potentially infectious material; post-exposure vaccination of susceptible persons in contact with persons at risk | Live vaccines are contraindicated in immunosuppressed patients (depending on the active substance and dose); seronegative persons should be vaccinated prior to planned immunosuppressive therapy or organ transplantation (if possible); post-exposure prophylaxis in susceptible immunocompromised individuals and pregnant women consists of passive immunisation with varicella-zoster immunoglobulin, if necessary in combination with antiviral chemoprophylaxis | Various live attenuated vaccines including monocomponent vaccines and combinations (MMRV); two vaccine doses are required |

Changes since 2020 are printed bold. Standard vaccination recommendations for children/adolescents and exclusive travel vaccinations and/or exclusive vaccinations for laboratory personnel are not included. A comprehensive list of available vaccines can be found on the website of the Paul-Ehrlich-Institut (https://www.pei.de/DE/arzneimittel/impfstoffe/impfstoffe-node.html)

Abbreviations:

TBE Tick-borne encephalitis

IPV Inactivated poliovirus

JAK inhibitor Janus kinase inhibitor

MMR Measles, mumps, rubella

MMRV Measles, mumps, rubella, varicella

PCV-13 13-valent pneumococcal conjugate vaccine

PCV-20 20-valent pneumococcal conjugate vaccine

PPSV-23 23-valent pneumococcal polysaccharide vaccine

STIKO Standing Committee on Vaccination

Tdap Combination of tetanus toxoid, diphtheria toxoid and acellular pertussis components

WHO World Health Organization

## **Table S2** Implementation of the indication vaccinations discussed in the text in children and adolescents

| **Immunocompetent children and adolescents** | **Immunocompromised/chronically ill children and adolescents** | **Available vaccines; vaccination schedules; comments** |
| --- | --- | --- |
| COVID-19 |  |  |
| No standard vaccination of children/adolescents | Vaccination of persons aged ≥ 6 months at increased risk of severe outcome due to underlying disease or congenital/acquired immunodeficiency | mRNA (Comirnaty^TM^, Spikevax^TM^) and protein-based vaccines (e.g. Nuvaxovid^TM^), each with the variant adaptation recommended by the WHO, age-specific licensing and dosage to be considered; baseline immunity is achieved by at least 3 antigen contacts, of which at least 1 vaccination; the indication groups mentioned receive an annual booster in autumn |
| Herpes zoster |  |  |
| No standard vaccination of children/adolescents | No vaccination recommended for immunocompromised children and adolescents |  |
| Influenza |  |  |
| No standard vaccination of children/adolescents | Vaccination of persons aged ≥ 6 months at increased risk of severe outcome due to underlying chronic disease or congenital/acquired immunodeficiency, including immunosuppressive treatment | Inactivated vaccines (e.g. Influsplit^TM^, Influvac^TM^); annual vaccination in autumn with a vaccine containing the current WHO-recommended antigen combination;  note: A live attenuated influenza vaccine (Fluenz^TM^) for nasal administration is available as an alternative for people aged 2-17 years, but is contraindicated in clinically manifest immunodeficiency |
| Pneumococci |  |  |
| Primary vaccination of infants aged ≥ 2 months (preterm infants also from chronological age ≥ 2 months) | Additional vaccination for children and adolescents (2-17 years) with congenital or acquired immunodeficiency, chronic diseases or with local risk factors for pneumococcal meningitis (e.g. cochlear implant, cerebrospinal fluid fistula) | 13-valent (PCV-13, Prevenar 13^TM^) and 15-valent (PVC-15, Vaxneuvance^TM^) conjugate vaccine; 23-valent pneumococcal polysaccharide vaccine (PPSV-23, Pneumovax^TM^ 23);  primary vaccination of full-term infants aged 2, 4 and 11 months (2+1); primary vaccination of preterm infants aged 2, 3, 4 and 11 months (3+1);  sequential vaccination of the above indication groups with PCV-13 or PCV-15, followed by PPSV-23 at an interval of 6-12 months; note: PCV-20 (Prevenar 20^TM^) has been licensed for use from 6 weeks of age since 03/2024; for the time being, the STIKO recommends primary vaccination with PCV-13/PCV-15 due to the lower immunogenicity of PCV-20; there is also no recommendation for the use of PCV-20 in immunocompromised children (as of January 2025) |
| Respiratory syncytial virus (RSV) |  |  |
| Single dose passive immunisation of neonates and infants | As recommended for immunocompetent children | Nirsevimab (Beyfortus^TM^), a monoclonal antibody directed against the viral F protein; infants born between April and September receive nirsevimab in the autumn before the start of the RSV season; neonates born during the current RSV season (October to March) receive nirsevimab as soon as possible (3-10 days after birth) |

Abbreviations:

PCV-13 13-valent pneumococcal conjugate vaccine

PCV-15 15-valent pneumococcal conjugate vaccine

PCV-20 20-valent pneumococcal conjugate vaccine

PPSV-23 23-valent pneumococcal polysaccharide vaccine

STIKO Standing Committee on Vaccination
